# Dietary supplementation of vitamin B1 prevents the pathogenesis of osteoarthritis

**DOI:** 10.1073/pnas.2408160121

**Published:** 2024-07-18

**Authors:** Shuying Shen, Yi Liang, Yuening Zhao, Ziang Hu, Youling Huang, Yizheng Wu, Yufei Liu, Shunwu Fan, Qingqing Wang, Peng Xiao

**Affiliations:** ^a^Department of Orthopaedic Surgery, Sir Run Run Shaw Hospital, Zhejiang University School of Medicine, Hangzhou 310016, China; ^b^Department of Gastroenterology, Sir Run Run Shaw Hospital, Zhejiang University School of Medicine, Hangzhou 310016, China; ^c^Institute of Immunology, Zhejiang University School of Medicine, Hangzhou 310058, China; ^d^Zhejiang Key Laboratory of Precision Diagnosis and Treatment for Lung Cancer, Yiwu 322000, China

**Keywords:** osteoarthritis, vitamin B1, macrophages

## Abstract

As the primary cause for chronic pain and disability in elderly individuals, osteoarthritis (OA) is one of the fastest-growing diseases due to the aging world population. To date, the impact of microenvironmental changes on the pathogenesis of OA remains poorly understood, greatly hindering the development of effective therapeutic approaches against OA. In this study, we profiled the differential metabolites in the synovial fluid from OA patients and identified the downregulation of vitamin B1 (VB1) as a metabolic feature in the OA microenvironment. In a murine destabilization of medial meniscus-induced OA model, supplementation of VB1 significantly mitigated the symptoms of OA. Cytokine array analysis revealed that VB1 treatment remarkably reduced the production of a pro-OA factor—C-C Motif Chemokine Ligand 2 (CCL2), in macrophages. Further evidence demonstrated that exogenous CCL2 counteracted the anti-OA function of VB1. Hence, our study unveils a unique biological function of VB1 and provides promising clues for the diet-based treatment of OA.

Osteoarthritis (OA) is a degenerative joint disease and the most common form of arthritis globally, which is a leading cause of disability among the elderly population. The typical symptoms of OA include pain and stiffness in the affected joints. The current treatment strategies for OA include regular exercise, acupuncture, and massage. Patients with severe OA have to undergo surgery, such as joint replacement.

Although the etiology of OA remains not fully understood, it is well recognized that the pathogenesis and progression of OA are closely associated with abnormal immune responses in the cartilage microenvironment (1). Apart from regulatory proteins, emerging evidence has underscored the crucial roles of immunoregulatory metabolites in various immune disorders (2). Compared to traditional medications, natural metabolites, many of which are derived from our daily diet, usually have advantages in terms of higher availability, proven safety, and lower costs. Therefore, the diet-based intervention of immunological diseases has been attracting increasingly more attention in recent years. However, the metabolic alterations in the cartilage microenvironment of OA patients and their potential effects on OA pathogenesis remain largely unexplored to date. Herein, we report a unique, anti-OA role of vitamin B1 (VB1), and the underlying mechanism was further explored.

## Materials and Methods

Detailed materials and methods are provided in *SI Appendix*.

## Results and Discussion

**Fig. 1.**
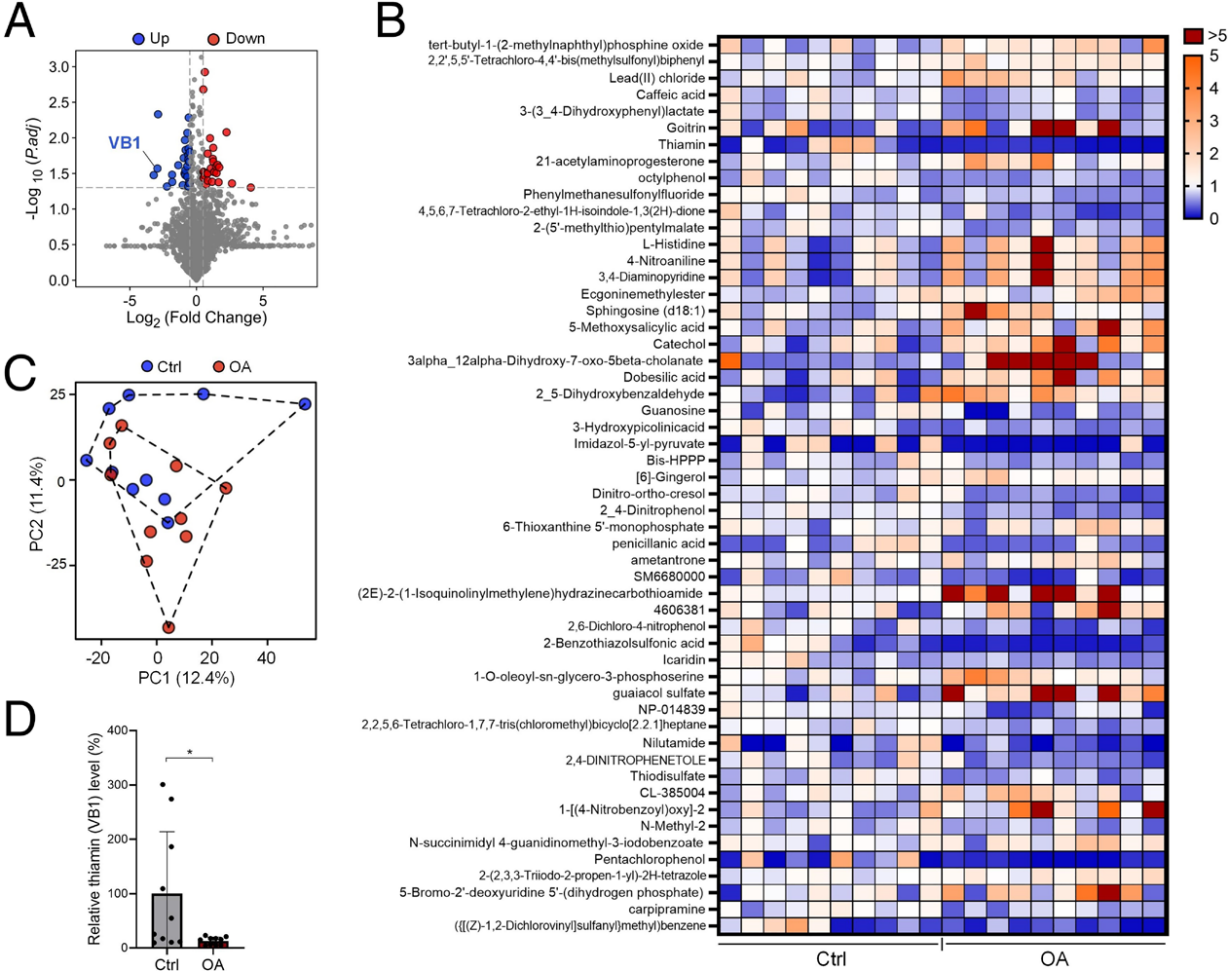
(*A*) Synovial fluid from OA patients and control subjects were subjected to untargeted metabolomics (n = 10/group). The differential metabolites were displayed in a volcano plot. (*B*) The differential metabolites from individual patients were shown in heatmap. (*C*) PCA was performed using differential metabolites between OA synovial fluids and control synovial fluids. (*D*) The level of VB1 for each individual was shown. *P < 0.05, unpaired, two-tailed Student’s *t* test.

To determine the metabolic changes in OA pathological microenvironment, we performed untargeted metabolomics using synovial fluid from OA patients and relatively healthy control subjects. A total of 26 downregulated and 27 upregulated metabolites (|Log_2_FC > 0.5 |, *P* < 0.05, OA vs. control) were identified (Fig. 1 *A* and *B*). Principal component analysis (PCA) demonstrated a clear separation between OA synovial fluids and control synovial fluids (Fig. 1*C*). Among these differentially produced metabolites, we noticed that VB1 (also known as thiamin), a water-soluble vitamin found in daily diet, exhibited the second largest decrease as assessed by fold change (Fig. 1 *A* and *D*), indicating the potential involvement of VB1 in OA pathogenesis.

**Fig. 2.**
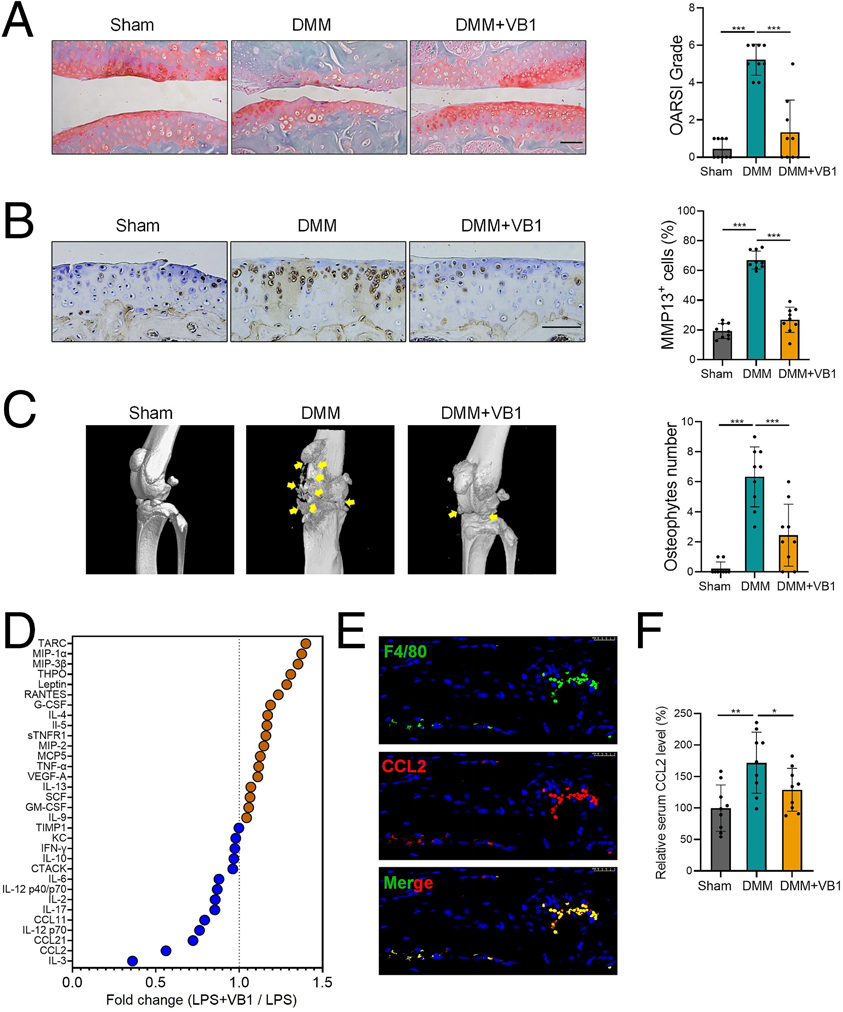
(*A*–*C*) DMM-induced murine OA model was established (n = 9/group), Safranin O/fast green staining (*A*) and immunohistochemical staining of MMP-13 (*B*) in the cartilages of sham or DMM mice. (Scale bar: 100 μm.) (*C*) Micro-CT images of murine knee joints. Yellow arrows indicate the osteophytes. (*D*) Mouse peritoneal macrophages were stimulated with LPS in the presence of PBS or VB1 for 16 h, the culture supernatants were harvested for cytokine array, fold changes of cytokines were shown. (*E*) Immunofluorescence staining showed the expression of CCL2 in F4/80^+^ synovial macrophages. (*F*) Serum levels of CCL2 in OA mice were evaluated by enzyme-linked immunosorbent assay (ELISA). *P < 0.05; **P < 0.01; ***P < 0.001, unpaired, two-tailed Student’s *t* test.

Therefore, we next established a murine destabilization of medial meniscus (DMM)-induced OA model with or without the administration of VB1. Safranin O/fast green staining showed that VB1 significantly prevented cartilage proteoglycan loss in OA mice (Fig. 2*A*). In addition, immunohistochemistry staining showed that VB1 significantly decreased the number of cells expressing MMP13, the primary enzyme responsible for cartilage degradation (Fig. 2*B*). Three-dimensional reconstruction micro-computed tomography (micro-CT) imaging demonstrated a significant reduction in osteophytes in OA mice administered with VB1 (Fig. 2*C*).

Our group has revealed the crucial roles of macrophages in inflammatory diseases (3–5). In addition, macrophage-mediated inflammation is closely associated with OA development (6). To investigate the potential impact of VB1 on the inflammatory activation of macrophages, we stimulated murine macrophages with lipopolysaccharides (LPS) in the presence or absence of VB1. The culture supernatants were subjected to cytokine array analysis. We observed that VB1 treatment markedly reduced the production of CCL2, a potent pro-OA mediator (7), in macrophages (Fig. 2*D*). Immunofluorescence staining further confirmed that CCL2 was predominantly expressed in synovial macrophages (Fig. 2*E*). Compared to control mice, serum levels of CCL2 were significantly elevated in OA mice, whereas VB1 treatment significantly reduced CCL2 levels in OA mice (Fig. 2*F*).

**Fig. 3.**
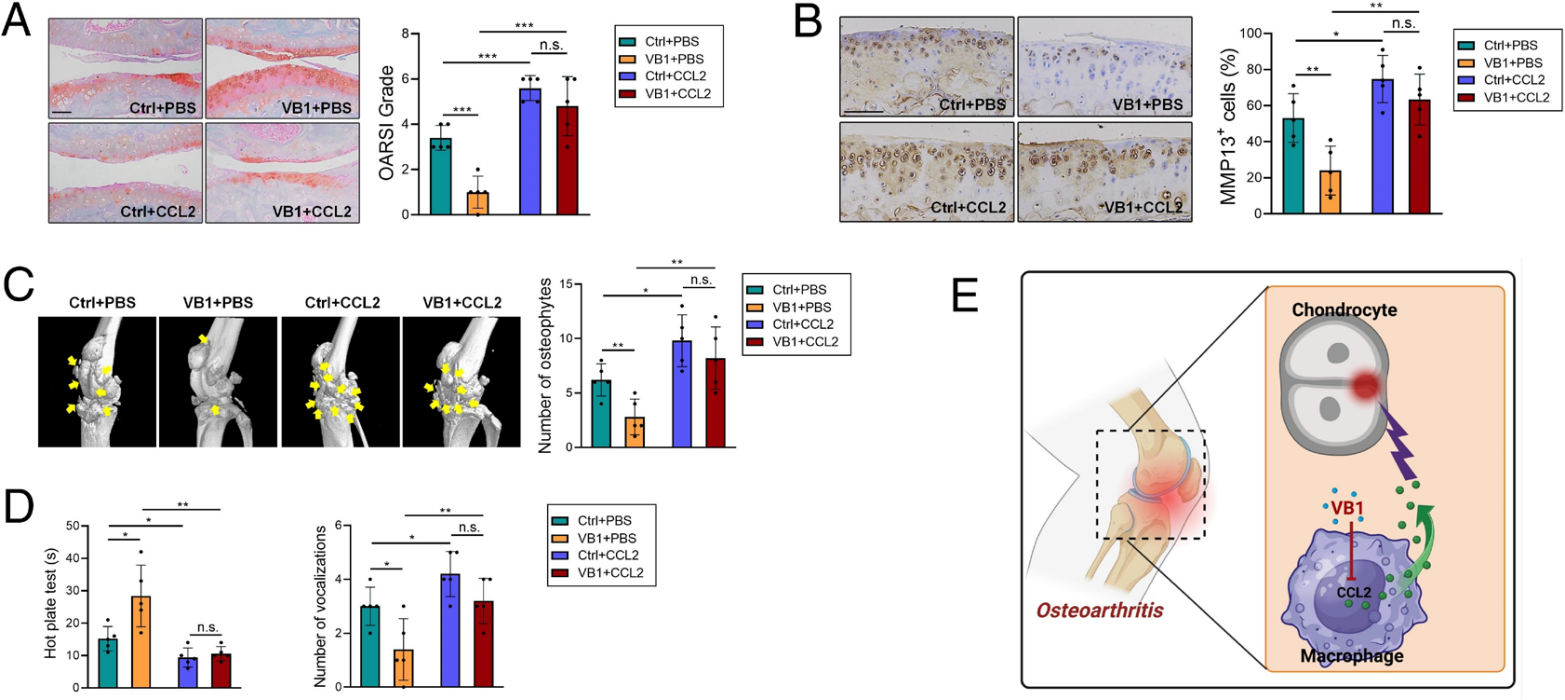
(*A* and *B*) DMM mice were administered with VB1, CCL2, or VB1+CCL2 (n = 5/group). Safranin O/fast green staining (*A*) and immunohistochemical staining of MMP-13 (*B*) were performed using murine cartilages. (Scale bar: 100 μm.) (*C*) Micro-CT images of murine knee joints. Yellow arrows indicate the osteophytes. (*D*) Hot plate test and knee extension test were performed to evaluate knee pain in OA mice. (*E*) Model describing the mechanisms of the anti-OA function of VB1. *P < 0.05; **P < 0.01; ***P < 0.001, unpaired, two-tailed Student’s *t* test.

In order to clarify whether VB1 mitigates OA development through CCL2 suppression, we coadministered recombinant CCL2 and VB1 into OA mice. The results showed that exogenous CCL2 challenge significantly exacerbated cartilage proteoglycan loss and increased the number of MMP13^+^ cells in OA mice (Fig. 3 *A* and *B*). Micro-CT scans also demonstrated that CCL2 treatment significantly increased the number of osteophytes (Fig. 3*C*). By performing hot plate test and knee extension test, we further observed that CCL2-treted mice exhibited more severe knee pain (Fig. 3*D*). The aforementioned pro-OA effects of CCL2 were more prominent in VB1-treated OA mice. Importantly, CCL2 administration dramatically blunted the comparative differences between control and VB1-treated OA mice in terms of cartilage proteoglycan loss, MMP13 expression, the number of osteophytes, and knee pain (Fig. 3 *A*–*D*). Taken together, VB1 prevents the pathogenesis of OA by reducing the production of CCL2 by macrophages (Fig. 3*E*).

The above data indicate that daily supplementation of VB1 may serve as a promising therapeutic strategy against OA and thus reduce the risk of disability and chronic pain in elderly people. VB1 is abundantly present in both natural and processed foods, and it is utilized as a nutritional supplement. VB1 deficiency causes various disorders such as beriberi, heart failure, tachypnea, muscle weakness, or neurologic implications (8). Previous study found that the levels of VB1 in the blood were increased after elective knee arthroplasty in OA patients (9). B-vitamin mixture comprising thiamine (VB1), pyridoxine (VB7), and cyanocobalamin (VB12) enhanced the analgesic effect of Diclofenac in OA patients who underwent total knee arthroplasty (10). Additionally, Fursultiamine, a VB1 derivative, was found to improve the therapeutic effect of glucosamine hydrochloride plus chondroitin sulfate in OA (11). In this study, we systemically profiled the metabolic changes in the OA microenvironment and identified VB1 as one of the most dramatically changed metabolites. The anti-OA function of VB1 is at least partially attributed to its capacity to suppress CCL2 expression. Compared to other chemokines, CCL2 plays a particularly crucial role in OA progression. OA patients exhibited increased CCL2 production in the synovial fluid compared to healthy controls (12). Mechanistically, CCL2 may exacerbate OA by mediating the recruitment of peripheral monocytes (13), inducing the expression of MMPs and VCAM-1 (12, 14), increasing the activity of collagenase (15), or promoting cartilage damage (16) or facilitating the production of inflammatory cytokines (17). As a natural and water-soluble vitamin, VB1 has relatively higher safety and lower costs compared to traditional anti-OA drugs, potentially enhancing patient compliance. Thus, increasing the intake of VB1-rich food (such as whole grains, beans, nuts, and fish) is a potentially beneficial dietary approach in OA patients.

Taken together, in this study, we identified a safe, natural nutrient that prevents the pathological processes of OA, thereby providing unique perspectives on dietary interventions for OA.

## Supplementary Material

Appendix 01 (PDF)

## Data Availability

All study data are included in the article and/or *SI Appendix*.
