# Supplementary material for: Dietary supplementation of vitamin B1 prevents the pathogenesis of osteoarthritis
Source: Proc Natl Acad Sci U S A. 2024 Jul 18;121(30):e2408160121. doi: 10.1073/pnas.2408160121 (PMC11287274; doi:10.1073/pnas.2408160121)
Supplement: Supplementary file 1 — Appendix 01 (PDF) [file pnas.2408160121.sapp.pdf]

Supplemental information

Materials and Methods

Clinical samples

The patients were classified into control and moderate to severe osteoarthritis groups based on the Kellgren-Lawrence (KL) grading of knee joints as we previously described (1). The control group comprised patients with KL grade 0 and 1, while the moderate to severe osteoarthritis group included patients with KL grade 3 and 4. Synovial fluids were obtained from patients with end-stage symptomatic knee OA at the time of total knee replacement surgery or from patients undergoing arthrocentesis. All samples were centrifuged at 12,000 rpm at 4°C for 5 minutes, and the supernatant was collected and stored at -80°C. The experiments were approved by the Medical Ethics Committee of Sir Run Run Shaw Hospital, Zhejiang University School of Medicine (#20211118-33).

Murine OA model

The DMM-induced OA model was established as we previously described (1, 2). VB1 (Sigma-Aldrich, St. Louis, MO, USA) was administered in drinking water at a final concentration of 1% during the experiment. 1 μg recombinant CCL2 (Peprotech, Cranbury, NJ, USA) was administered by intra-articular injection every week, starting from 14 days after DMM surgery. Mice were sacrificed two months post DMM surgery. Animal experiments were performed according to protocols approved by the Zhejiang University Institutional Animal Care and Use Committee.

Metabolomics analysis

Metabolomics analysis was performed by Nanjing Jiangbei New Area Biopharmaceutical Public Service Platform Co., Ltd (Nanjing, China). The procedures are as follows: 100 μl samples were added with 400 μl extraction buffer (methanol:acetonitrile=3:1, pre-colded at -40°C), vortexed for 5 minutes, ultrasound for 15 minutes, and incubated at 4°C for hours. The samples were then centrifuged at 12000 rpm for 15 minutes, followed by vacuum concentration. Afterwards, 100 μl 50%

methanol solution was added to redissolve the samples, vortexed for 3 minutes, and centrifuged at 12000 rpm for 15 minutes. The supernatants were loaded into an Ultra High Performance Liquid Chromatography (UHPLC) system (Thermo Fisher Scientific), and were separated using HSS T3 column (1.7μm, 2.1 mm× 150 mm, column temperature: 40°C; flow rate: 0.3 mL/min). Mobile Phase A: formic acid in water (0.1%), B: formic acid in methanol (0.1%), C: acetic acid in water (0.05%), D: acetic acid in methanol (0.05%). The mass spectrometry data were collected on a Thermo QE HF-X mass spectrometer (Thermo Fisher Scientific).

Macrophage treatment

Murine peritoneal macrophages were isolated and cultured as we previously described (3). Macrophages were stimulated with 1 μg/ml LPS (Sigma-Aldrich) in the presence of VB1 or PBS. Sixteen hours later, culture supernatants were collected and centrifuged at 1000 g for 15 minutes. The supernatants were then subjected to cytokine array.

Cytokine Array

Cytokine array membranes from RayBiotech (Norcross, GA, USA) were blocked in blocking buffer for 30 minutes, then incubated with macrophage supernatants overnight at 4°C. On the next day, the supernatants were discarded. The membranes were washed with washing buffer, followed by incubation with biotin-conjugated antibodies for 2 hours at room temperature. Thereafter, the membranes are washed with washing buffer, reacted with streptavidin-conjugated fluor at room temperature, then scanned on an Axon scanner (Molecular Devices, San Jose, CA, USA). The intensities of signals were quantified using densitometry.

ELISA

The levels of CCL2 in mouse serum were evaluated using ELISA kit from Peprotech according to the manufacturer's protocol.

Immunohistochemistry, Safranin O/fast green staining, immunofluorescence, and micro-CT analysis were all conducted as we previously described (1, 2).

Hot plate test and knee extension test were conducted as we previously described (2).

Statistical analysis

Data were analyzed using GraphPad Prism 8.0 Software (GraphPad Software, Inc.) and were presented as mean ± standard deviation. Unpaired, two-tailed Student's t test was adopted to compare the differences between two groups.

References

1. Wu Y, et al. (2023) Metabolite asymmetric dimethylarginine (ADMA) functions as a destabilization enhancer of SOX9 mediated by DDAH1 in osteoarthritis. Sci Adv 9(6):eade5584.

2. Shen S, et al. (2021) circPDE4B prevents articular cartilage degeneration and promotes repair by acting as a scaffold for RIC8A and MID1. Ann Rheum Dis 80(9):1209-1219.

3. Xiao P*, et al.* (2019) Phosphatase Shp2 exacerbates intestinal inflammation by disrupting macrophage responsiveness to interleukin-10. *J Exp Med* 216(2):337-349.
